# Interprofessional collaboration and barriers among health and social workers caring for older adults: a Philippine case study

**DOI:** 10.1186/s12960-021-00568-1

**Published:** 2021-04-19

**Authors:** TJ Robinson T. Moncatar, Keiko Nakamura, Kathryn Lizbeth L. Siongco, Kaoruko Seino, Rebecca Carlson, Carmelita C. Canila, Richard S. Javier, Fely Marilyn E. Lorenzo

**Affiliations:** 1 Department of Global Health Entrepreneurship, Division of Public Health, Tokyo Medical and Dental University, Tokyo, 113-8519 Japan; 2 Department of Health Policy and Administration, College of Public Health, University of the Philippines Manila, 1000 Metro Manila, Philippines; 3 World Health Organization Collaborating Centre for Healthy Cities and Urban Policy Research, Tokyo, 113-8519 Japan; 4 College of Nursing, University of the Philippines Manila, 1000 Metro Manila, Philippines; 5 Institute of Global Affairs, Tokyo Medical and Dental University, Tokyo, 113-8519 Japan

**Keywords:** Older adults, Interprofessional collaboration, Health and social care, Service delivery, Human resources for health, Qualitative research, Philippines

## Abstract

**Background:**

There is limited information on how the barriers to interprofessional collaboration (IPC) across various professionals, organizations, and care facilities influence the health and welfare of older adults. This study aimed to describe the status of IPC practices among health and social workers providing care for older adults in the Philippines; investigate the perceived barriers to its implementation and perceived effects on geriatric care; and identify possible solutions to address the barriers limiting collaborative practice.

**Methods:**

A case study approach was utilized employing 12 semi-structured in-depth interviews and 29 focus group discussions with care workers from selected primary health care units, public and private hospitals, and nursing homes that are directly involved in geriatric care delivery in two cities in the Philippines. Overall, 174 health and social workers consented to participate in this study. All interviews were audio-recorded and transcribed verbatim. An inductive thematic analysis using NVivo 12® was used to identify and categorize relevant thematic codes.

**Results:**

Interprofessional geriatric care provided by health and social workers was observed to be currently limited to ad hoc communications typically addressing only administrative concerns. This limitation is imposed by a confluence of barriers such as personal values and beliefs, organizational resource constraints, and a silo system care culture which practitioners say negatively influences care delivery. This in turn results in inability of care providers to access adequate care information, as well as delays and renders inaccessible available care provided to vulnerable older adults. Uncoordinated care of older adults also led to reported inefficient duplication and overlap of interventions.

**Conclusion:**

Geriatric care workers fear such barriers may aggravate the increasing unmet needs of older adults. In order to address these potential negative outcomes, establishing a clear and committed system of governance that includes IPC is perceived as necessary to install a cohesive service delivery mechanism and provide holistic care for older adults. Future studies are needed to measure the effects of identified barriers on the potential of IPC to facilitate an integrated health and social service delivery system for the improvement of quality of life of older adults in the Philippines.

**Supplementary Information:**

The online version contains supplementary material available at 10.1186/s12960-021-00568-1.

## Background

As the older population aged 60 and above increases, several age-related health problems will lead to significant financial, social, and psychological burden for patients, families, and healthcare systems. These will require coordination of ongoing care, expertise, and support from human resources for health (HRH), community-based workers, and social welfare providers [1–3]. In the Philippines, primary care networks render basic services such as health education, promotion, and primary care to the aging population. But due to lack of HRH, referral systems to higher care facilities such as public or private hospitals are resorted to for further patient evaluation and management [4]. It is fortuitous that several targeted policies and programs were initiated for older adults through landmark legislations in recent years [5]. There are numerous policies on paper and a multitude of geriatric services provided in specific care settings from each profession or organization. However, a significant number of older Filipinos still experience growing unmet needs related to financial, health care, social services, and family support [6, 7].

Meeting the unique health and social services needs of older adults is beyond the expertise of any single HRH, profession, or organization [8, 9]. The care needed by older adults is diverse, complex, and labor intensive. It encompasses different workforce from community health centers, hospitals, and nursing homes required to be well-skilled and highly committed to deliver quality, comprehensive, and effective geriatric care services [10]. Enhancing collaboration and communication among various professionals across different providers, organizations, and sectors is one strategy to optimize resources, improve quality and safety, and bridge healthcare fragmentation [11, 12]. To this end, interprofessional collaboration (IPC) works to ensure cohesion, consistent dialogue, greater resource efficiency, improved standards of care through limiting duplication and gaps in service provision, delivery of holistic services, and better continuity of care [13, 14]. IPC is defined as a partnership between a team of health providers and a client in a participatory collaborative and coordinated approach to shared decision-making around health and social issues, which includes communication and decision-making, enabling a synergistic influence of grouped knowledge and skills [15]. Other interchangeable term often used to describe IPC is collaboration, which occurs when two or more entities mutually and beneficially work together to produce a desired and shared outcome [16]. Collaborative practice is an efficient, effective, and satisfying way to offer health care services and another term depicting interprofessional teams [17]. This happens when multiple health workers from different professional backgrounds work together with patients, families, carers, and communities to deliver the highest quality of care across care settings [14]. Adoption of an interdisciplinary, multi-level, and multi-stakeholder approach is crucial to facilitate effective HRH management building a resilient and innovative workforce [10]. Further, IPC is an intervention that involves members of more than one care sector or organization interacting with other key individuals (i.e., patients, families) with the explicit purpose of enhancing collaboration to improve access to care and patient health outcomes [11, 18]. A previous study also revealed that IPC practice led to improved medication use, decreased length of hospital stays, and total hospital charges [19]. IPC, along with organizational culture, is a predictor of 35% of job satisfaction among health care teams [20]. Implementation of an inclusive and comprehensive IPC strategy is a key HRH solution across health and other related service sectors that is fundamental to the implementation of Universal Health Coverage (UHC) [21].

Although IPC has been shown empirically to improve professional practice, delivery of care services, and health outcomes [14, 19, 22], especially for rapidly aging societies [23, 24], several interpersonal factors, including power and hierarchical differences among individuals, may inhibit its implementation [22]. In contrast, cordial interaction, respectful communication, and acknowledgment of others’ contributions resulted in satisfaction among interprofessional teams [25]. Organizational structure and systemic conditions such as professional, educational, and social environment within and outside the care setting play critical roles in the practice of teamwork and collaboration among care professionals, but have received less attention [17]. A previous qualitative study also highlighted the dearth of empirical evidence on organizational culture and systems [26]. While different healthcare systems have been experimenting with IPC to improve the wellbeing of older adults and their daily functioning [14, 27], evidences are primarily from high-income countries [19, 22, 24, 28]. Little is known about the distinct challenges to implementation that may be faced by healthcare systems in low- and middle-income countries (LMICs) [29].

In the Philippines, IPC as a strategy to alleviate human resource challenges is currently gaining traction although research on its implementation is still limited [30]. Human resource issues on geriatric care have yet to gain sufficient research attention [10]. There is also a paucity of evidence about how existing barriers, which limit IPC implementation, may impact the wellbeing of older adults [17]. The World Health Organization (WHO) identified guiding principles and solutions to foster implementation of interprofessional education and collaborative practice, but are context dependent and not generalizable [31]. It is necessary to have a greater understanding of possibilities for IPC implementation across diverse individuals and organizations working at tertiary, secondary, and primary levels care, in a variety of national settings to ensure a responsive health care delivery system that addresses the unmet needs of older adults. Hence, in this study, we aspired to further this important area of inquiry by asking the following questions: (1) What is the status of IPC in the Philippines and how does it vary across the individuals and organizations that render care and services for older adults?; (2) What are the perceived barriers to IPC among health and social workers in different settings and how do these contribute to quality geriatric care?; (3) What are the potential solutions to addressing the barriers to IPC implementation in these settings?

## Methods

The present study is a component of a larger mixed-methods research project that aimed to develop a competency-based in-service interprofessional education (IPE) training program for health and social workers to enhance IPC and improve the quality of care for older adults. This study provides information on HRH experiences and practice of IPC for geriatric care that will contribute to the context specific design of the IPE training program development that will be implemented in the Philippines and other Southeast Asian countries. Moreover, given the paucity of information on IPC implementation particularly from LMICs, the evidences in this study can add in further strengthening the global call for action to facilitate IPE and collaborative practice towards health systems and outcomes improvement.

### Research design

This study utilized a qualitative case study methodology [32] founded on social constructivism theory that posits that the knowledge and understanding of an individual is shaped by their interactions within a specific social context [33]. Qualitative methods are designed to elicit participant’s own perspectives and experiences in a detailed and in-depth manner expressly to explore the relationship of variables in the care of older persons [34]. To explore the status of, and perceived barriers to, IPC in health and social care settings, in-depth interviews or focus group discussions (FGDs) (ranging from two to 15 participants), were held with workers directly involved in geriatric care. The use of FGD with homogenous groupings of health and social care workers per health facility was the primary method of qualitative inquiry in this study. This was performed to assess on the mutual agreements and disagreements, ensure comfort, openness, and higher degree of interaction among the participants. However, in-depth interview was also utilized due to uncontrolled circumstances (i.e., only one available participant or working in a specific facility during schedule of inquiry), but enabled the team to further discuss and probe on sensitive issues that may be difficult to obtain or missed during FGDs of similar profession.

To ensure sampling breadth, a variety of facilities were first selected. Secondarily, care workers were recruited from these selected health facilities. The recruitment of key study participants in different settings was paramount in gathering participation from health workers, who are tasked with preventive, curative, and rehabilitative interventions, and social workers who offer welfare and support services for older adults.

### Study settings

Two cities located within the Greater Manila Area (GMA) in the Philippines were purposively selected due to their significant proportion of older adults to total population. Moreover, these cities have a substantial number of tertiary hospitals and nursing homes catering directly to the needs of their older population. Health and social workers implementing programs and services for older adults at the primary care level (i.e., health centers) were solicited for participation. In both areas, city officials recommended one public and two private tertiary hospitals, as well as three nursing homes to participate in the study.

### Sampling and recruitment

The research team secured a formal mandate from local government authorities, specifically mayors and other city officers, to contact staff working at the primary care level for participation in the study. Local government staff also identified and recommended hospitals and nursing homes providing care to older adults for inclusion. Consent was then solicited after providing both written and oral descriptions of the project and its aims. Relevant directors and managers who agreed to participate in the research distributed the study description to staff meeting the inclusion criteria: being a currently employed health or social worker and being an established care provider for older adults. Interviews and FGDs were only conducted after potential study participants signified their consent. Furthermore, other key offices actively providing care for older adults were subsequently identified by city officials and were snowballed into the study.

The research team conducted a total of 12 in-depth interviews and 29 FGDs, involving 174 participants. These were conducted, at the primary care health facilities, public and private tertiary hospitals, as well as in nursing homes throughout January and February 2019. All the available health and care workers during the duration of data collection were included in the study sample. Multiple key professionals involved in the implementation of programs and services for older adults such as physicians, nurses, and social workers were interviewed to determine the extent of collaborative practice [14]. In addition, non-professionals who were trained, and working in their respective areas such as community health workers, nutritionists, and community leaders tasked with connecting older adults to various health and social services were also included in this study. Auxiliary staff such as nursing assistants supporting healthcare professionals in hospitals, together with caregiver’s in-charge of aiding older adults with their activities of daily living (ADL) in nursing homes, were also interviewed. Inclusion of workers from different backgrounds and care settings will reflect the network of geriatric care delivery for older adults in selected areas of the Philippines [4]. The detailed characteristics of all individuals who participated in the qualitative interviews are shown in Table 1.

**Table 1:** Profile of health and social care worker participants by care setting

Characteristics	Primary care		Public hospital		Private hospital		Nursing home		Total	
	*n* = 122	(%)	*n* = 18	(%)	*n* = 15	(%)	*n* = 19	(%)	*n* = 174	(%)
Gender										
Female	103	84.0	10	56.0	7	47.0	16	84.0	136	78.0
Male	19	16.0	8	44.0	8	53.0	3	16.0	38	22.0
Age (years)										
20–39	19	16.0	10	56.0	13	87.0	16	84.0	58	33.0
40–59	64	52.0	8	44.0	2	13.0	2	11.0	76	44.0
≥ 60	39	32.0	–	–	–	–	1	5.0	40	23.0
Occupation										
Physician/specialist	6	5.0	5	28.0	2	13.0	–	–	13	7.0
Dentist	–	–	1	6.0	–	–	–	–	1	1.0
Nurse	9	7.0	5	28.0	4	27.0	2	11.0	20	11.0
Midwife	12	10.0	–	–	–	–	–	–	12	7.0
Rehabilitation therapist	1	1.0	3	17.0	4	27.0	2	11.0	10	6.0
Nutritionist	8	7.0	–	–	–	–	–	–	8	5.0
Social worker	3	2.0	2	11.0	1	7.0	–	–	6	3.0
Nursing assistant	–	–	2	11.0	4	27.0	–	–	6	3.0
Elderly clinic assistant	1	1.0	–	–	–	–	–	–	1	1.0
Caregiver	–	–	–	–	–	–	15	79.0	15	9.0
Community health worker	39	32.0	–	–	–	–	–	–	39	22.0
Community nutritionist	14	11.0	–	–	–	–	–	–	14	8.0
Community leader	29	24.0	–	–	–	–	–	–	29	17.0
Length of service										
< 1 year	6	5.0	2	11.0	4	27.0	8	42.0	20	11.0
1–5 years	34	28.0	5	28.0	9	60.0	5	26.0	54	31.0
6–10 years	34	28.0	7	39.0	1	7.0	5	26.0	53	31.0
> 10 years	48	39.0	4	22.0	1	7.0	1	5.0	47	27.0
Area of work assignment										
City A	59	48.0	–	–	6	40.0	7	37.0	102	59.0
City B	63	52.0	18	100.0	9	60.0	12	63.0	72	41.0

– no participants included

### Data collection and procedure

The in-depth interviews and FGDs were primarily conducted by two researchers (TRTM and KLLS) who took turns in facilitating and note-taking. A semi-structured interview guide was used during data collection, covering topics such as working experiences, practice of interprofessional collaboration and evaluation, observed characteristics of older adults, awareness and experience related to gaps between patient needs and services rendered, as well as difficulties older adults face in accessing health and social welfare services (see Additional file 1). Interviews were conducted in rooms with only the researchers and participants present to ensure privacy. All interviews lasted approximately 60 to 90 min and were audio-recorded with the participants’ consent. Three researchers (CCC, RSJ, and FMEL) supervised all data collected to ensure quality and robustness.

### Data analysis

Interviews were transcribed verbatim and translated to English (TRTM and KLLS) with support from trained research assistants. Analysis began during data collection in order to determine emerging patterns and identify questions to further probe during subsequent interviews (TRTM and KLLS). Crosschecking of the transcripts with field interview notes, reflective memos, and discussion between the two primary researchers were conducted to achieve consensus. Data management was aided by NVivo 12® (QSR International, Burlington, MA, USA) and an inductive thematic analysis was used to guide in the identification of descriptive codes [35]. This process was repeated across all transcripts until analytical saturation was achieved [36]. These initial findings were then presented and discussed with a qualitative methods specialist (RC), which led to refinement of results during analysis. Afterwards, senior research members (KN, KS, CCC, RSJ, and FMEL) reviewed and validated the themes and then presented the analysis to selected interview participants and key stakeholders to gain feedback and ensure trustworthiness of the findings, which contributed to rigor of the results [37].

### Ethical considerations

Three research Ethics Boards approved this study’s protocol: the World Health Organization Research Ethics Review Committee [ERC.0003093], Tokyo Medical and Dental University Medical Research Ethics Committee [M2017-232] and the Philippine Department of Health Single Joint Research Ethics Board [SJREB-2018–21]. All study participants were provided written informed consent forms along with information about the study and an opportunity to ask questions prior to interviews or decline to participate. The research team strictly observed confidentiality and anonymity of participants’ identities and responses.

## Results

The key findings of this study are categorized according to the following sections: (1) Informal IPC, (2) Barriers to IPC implementation aggravating unmet needs, and (3) Potential solutions. Specifically, it seems that IPC for geriatric care among health and social workers in the Philippines is largely informal and consequently, ineffective. Several barriers were identified as primary causes limiting the practice of formal IPC. These were perceived by the participants as adversely affecting service delivery resulting in worsened health outcomes and lowered quality of life for older adults. A strong and committed governance mechanism was seen vital to address these barriers enabling collaborative practice to function.

## Informal IPC

In all interviews conducted across multiple workers in different care settings, participants described IPC as occurring only on an ad hoc and inconsistent basis, lacking formal structures or guidelines for IPC. This ad hoc communication and collaboration varied in frequency, intensity, participants, and nature. At the primary care level, partnership with other health and social care personnel was perceived to be lacking, limited to patient referrals to other professionals or facilities. These informal meetings were rarely conducted, and participants believed that these were largely underutilized involving primarily administrative concerns with little attention to the needs of older adults. Also, participants observed that feedback of patient status occurred only when convenient such as during informal meetings or communication among workers, but lacked structure.

Similar to these findings, participants from public and private hospitals reported that informal sharing of information was largely contained within specific professions (i.e., nurse-to-nurse only). Patient rounds and medical charts, considered legal documents in hospitals, were perceived as the primary, although limited, form of communication between occupations.

*“Communication and collaboration only happen through reading of papers. It starts and ends with charts. There is a transfer of information through patient’s records and not on a personal level. There is no exchange of thoughts on what happened to the patient.” (Rehabilitation therapist, Public hospital, FGD)*

IPC was perceived to be the most lacking in nursing home settings.

*“For me, it is like zero (IPC meetings). Sometimes, we always handle the same patients. We do our own work without communication and collaboration.” (Caregiver, Nursing home, In-depth interview)*

Nursing home caregivers were primarily focused on delivering the basic needs for a specific patient. Ad hoc communication was limited to managers and minimized the involvement of such frontline staff. The lack of possibilities for collaboration across various practitioners and care settings was viewed negatively by participants and perceived to be a problematic limitation that needs to be addressed.

## Barriers to IPC implementation aggravating unmet needs

Participants attributed the current lack of IPC in health and social service delivery for older adults to a confluence of barriers at the personal, organizational, and particularly systemic levels. Table 2 shows a summary of barriers identified by participants. Most critically, barriers to IPC were identified by participants as lowering the quality of care service delivery and exacerbating the increasing unmet needs of older adults.

**Table 2:** Barriers to IPC implementation across individuals, organizations, and care settings

Themes	Sub-categories
Personal values and beliefs	Egotism vs self-depreciation Lack of trust and respect Neglect and closed-mindedness Unfamiliarity of roles and services
Organizational resource constraints	Health human resource constraints Financial difficulties Logistical challenges
Silo systems care culture	Working independently Lack of emphasis on older adults Absence of leadership Absence of structure, standards, and policies

### Personal values and beliefs

Interpersonal issues generally pertaining to divergent personal values and beliefs were described as one barrier to IPC implementation, particularly by personnel at the primary care and nursing home levels. Caregivers, nursing assistants, and community health workers described the impact that staff hierarchies, as well as pride among other health professionals, had on teamwork and communication. These workers tended to undervalue themselves as frontline staff and felt it difficult to relay information believing they were *“voiceless”* and *“did not have the right”* to speak up, given their position within the larger healthcare team. In particular, some believed that professionals with a higher status such as doctors and nurses distrusted their expertise and abilities.

*“Other health workers such as nurses do not talk to us because they think that they are higher than us as we are only caregivers. Even as we approach them properly, they will just ignore us.” (Caregiver, Nursing home, FGD)*

This hierarchy was also reflected in the belief that hospital practitioners preferred to manage cases alone and ultimately found collaboration with others unnecessary.

*“We cannot tap and work with others (in the hospitals) because they do not like this kind of set-up (collaboration between primary care level and hospitals). Some people (doctors and specialists in hospitals) think that they can handle and treat the patients on their own. They do not need anybody else.” (Physician, Primary care level, In-depth interview)*

Some participants also argued that the lack of awareness of roles, abilities, and services of other care workers and facilities was quite profound. This resulted to potential teammates limiting their expectations of staff and organizations to specific capacities because of unfamiliarity and insufficient personal socialization which in turn hamper IPC implementation.

Multiple workers in different care settings believed that presence of professional hierarchy contributed to inadequate knowledge regarding patient status or interventions for care and treatment resulting to a decline in the quality of both health and social services detrimental to the health and welfare of older adults. In one nursing home, the lack of shared information related to patient conditions as the result of staff power differentials was eventually related to prolonged and serious pressure ulcers among residents.

*“We have difficulties in communication not just with the heads but other caregivers as well. The heads have an attitude problem. Sometimes, I will communicate with them and they will say negative things about me. Caregivers cannot talk or make suggestions. It is hard to request things. If we talk, we are still wrong. It is still the heads that should be followed. We are just workers. Proper communication with everyone is a problem and because of this, most bed-ridden older adults have many bedsores as no one is being informed of the patient condition.” (Caregiver, Nursing home, In-depth interview)*

Although participants identified personal values and beliefs as barriers, they were also recognized as vital enablers of ad hoc forms of communication currently practiced. Adherence to fairness despite their high status was noted among doctors while those at the primary care level, such as nurses and community health workers believed that receptiveness, commitment, and appreciation for collaboration all helped to facilitate informal communication among staff. Also, several practitioners believed that having a “*mind and heart for older adults*,” or an innate desire to provide quality care, was necessary to ensure communication with co-workers that could positively benefit this population.

### Organizational resource constraints

All participants strongly expressed that conditions within care settings have a profound influence on the lack of formal IPC. Notably, the presence of organizational resource constraints was more commonly cited as a key factor hindering teamwork and collaboration than the types of personal barriers described above. Various constraints on human resources were mentioned, such as staff shortages along with the limited number of specialists for geriatric care. In hospitals, participants explained that additional remuneration would be necessary to motivate professionals to collaborate as this was viewed as an additional task on top of their routine work requiring added financial incentives. As caseloads were increasing, hospital staff also felt there was inadequate time to perform interprofessional team meetings to provide quality care for all patients.

*“I am the only geriatrician here (hospital). So, who will they go to? Only me. I still have a social life. I am sorry. I do not have the time. It is difficult to gather people (to collaborate).” (Physician, Private hospital, In-depth interview)*

Human resource constraints were exacerbated by a lack of funds within organizations resulting not only in job vacancies and an inability to meet staffing requirements, but a lack of necessary diagnostic and treatment technologies such as cardiac monitors and medications.

*“Patient need is a challenge. How can we collaborate if we lack manpower, budget, and equipment? If the resources are inadequate, how will the unit work?” (Nurse, Public hospital, FGD)*

Some participants also felt that other additional requirements necessary to perform collaboration, such as available meeting spaces and working arrangements culturally common in the Philippines (e.g., refreshments during meetings), were also absent. These organizational constraints were seen to significantly hinder IPC implementation.

Consequently, several participants observed that the lack of organizational resources that inhibit IPC practice among health and social care workers resulted to inadequate dissemination of information about the specific services provided by each workers and offices such as social pension, medical assistance, or free medications and diagnostics offered at the community level, limiting older adults’ opportunities to access them and lowering overall quality of care.

The participants also shared that delays in accessing geriatric care services were also caused by human resource constraints. At the primary care level, this caused a delay in visiting older adults in their homes, while within hospitals and nursing homes, heavy workloads and staff shortages also resulted in insufficient time available to attend to patient needs. Participants at the primary care level and hospitals also frequently referred to lengthy procedures and waiting times that older adults generally experienced when attempting to access services.

*“You (older adult) have to go through a long process. The process takes a long time before you can request something for free. By the time the supply arrives, the senior citizen is dead already. Things would be better without the lengthy process. For example, if there is a senior who needs a wheelchair, I can just go (and communicate) with the social welfare office. That would be useful.” (Community health worker, Primary care level, FGD)*

### Silo systems care culture

The principal barrier to IPC was described as systems factors external to individual professionals and institutions. Particularly, all participants cited that a disjointed health and social care system was the greatest limitation of IPC. At the primary care level, services for older adults such as the provision of social pensions, financial and medical assistance, and the distribution of medications are designated primarily to social welfare units. Across hospitals and nursing homes, staff such as social workers were typically not involved in routine care delivery (i.e., patient rounds, shift handover) and were perceived by medical staff as out-of-place in hospitals. Health and social welfare function were found to be largely disparate from one another with primary care facilities and hospitals providing services for older adults separately, and in contact only during the process of patient referral.

*“We have a social worker, but we do not usually work with them. They have their own process and procedures. In addition, for example, communications happening between physical therapists and other therapists or doctors to therapists only. You can see in the doctor’s orders that the system is that workers independently perform interventions for patients.” (Nurse, Private hospital, In-depth interview)*

Furthermore, several participants strongly perceived that older adults are not considered health care priorities. This perception results in a paucity of training and capacity building focused on properly and effectively addressing older adult needs. A champion, focal person or leader, who could push for collaboration was also felt to be missing. In hospitals, participants stated that without guidelines it was difficult to collaborate or communicate across such a siloed system.

*“We need a standard operating procedure. Right now, we are just implementing services on our own.” (Social Worker, Public hospital, FGD)*

Ultimately, all participants argued that the absence of guidelines and standards for facilitating an integrated health system was seen to profoundly limit collaboration.

Siloed systems across all settings were strongly identified as causes of inadequate dissemination of information about care services and interventions subsequently reducing likelihood of older adults receiving them. In hospitals, participants identified that lack of collaboration across health and social care units, as well as other care settings led to lack of awareness about the availability of current services such as social support or counseling, also adversely affecting the overall quality of geriatric care.

*“There are no meetings with the Office of the Senior Citizen Affairs (OSCA, association of older adults ensuring government provision of healthcare and benefits), but there should be. Our link (to care facilities) is only during referral. Now, not all the senior citizens in the community know what OSCA provides and we are not able to share it with them every time they are in the hospital for outpatient consultation or confinement in the wards. Information is not cascaded to them.” (Social worker, Public Hospital, FGD)*

Participants argued that organizational siloes led directly to disparity in services for older adults. The lack of structured collaboration pushed health and social care workers to implement services disparately which tended to result in delayed and insufficient care delivery. In particular, the lack of collaboration between primary care facilities and hospitals led directly to delayed provision of key services for older adults such as medical and financial assistance or referrals.

Further, participants at the community level identified that there was a lack of uniformity of programs which also limited the ability of older adults to access specific needed services. Several older adults failed to receive social pensions or free medication due to a lack of collaboration and communication among the concerned organizations. Inequitable distribution of care was observed to increase health care expenses in general. Participants felt that wealthier patients could easily access social services compared to poor patients who need these services more. Wealthier patients have higher financial capacity and better service information, while poor patients experience limited care coverage and are exposed to inefficient intervention targeting. They observed that poorer older adults have higher financial burden and other physical barriers contributing on their difficulty to access services resulting to unfavorable conditions.

*“We are not involved with them (OSCA, social welfare office). We have our data. The senior citizen officers also have their data. There are a lot of elderly who need help. That is where they should focus because there are a lot [of older persons]. When it comes to getting the money (social pension), you see many who are not able to get it. There are more poor elderly but still not able to access their pensions.” (Community nutritionist, Primary care level, FGD)*

These types of delays and access difficulties for older adults in different care settings were also viewed to result in patient dissatisfaction in the quality of care and services provided.

Several participants also felt that working within a siloed care system resulted to redundancies in care and unnecessary duplication of interventions. At the primary care level, social workers observed that programs for older adults were often duplicated or performed improperly due to a lack of communication between organizations. For example, some older adult patients received the same medication from different healthcare facilities. Although institutional funds and supplies were frequently limited, some patients also received similar interventions from different units. In addition, the lack of communication between staff and institutions resulted in staff needing to recreate the documents required for older adults to access social welfare services.

*“The medical certificate we give to the elderly goes to city officers and social welfare workers to ask for financial support. The certificate expires every 3 months and then I must make them again. If I gave out 100 medical certificates, I must remake all those 100 medical certificates again. That is stupid but the city wants to redo everything again. The elderly is at loss since they must go back again and again. They don’t even have money to commute.” (Physician, Primary care level, FGD)*

Many participants perceived that the lack of communication and collaboration due to a disjointed health and social care system resulted in inefficient use of scarce resources limiting the accessibility of needed care services and contributed to additional out-of-pocket costs exacerbating the burden on older adults.

## Potential solutions

Despite the presence of these barriers and their potential impact on the wellbeing of older adults, several participants from all health facilities strongly expressed a desire to collaborate across individuals, organizations, and care settings.

*“We need to integrate the system and gel them together. We are different capable people. We are good individually, but we are better when working together. We need to have teamwork.” (Physician, Public hospital, FGD)*

Participants argued that an integrated health and social care system is necessary to improve service delivery, patient outcomes, as well as address resource constraints. Hospital staff in particular described the need to strengthen and empower the primary care level to provide preventive strategies and surveillance, as an approach to decrease the number of patients admitted to hospitals with complex morbidities, in addition to fostering stronger ties between the health sectors.

*“You cannot say that you drop this one (medicines). What would the other doctor feel? If the patient has co-morbidities, they have 18 to 20 medications. The kidneys or liver of the patients get destroyed; chances are after two weeks they are here again. The patient is pitiful. Their organs get treated but do the patients get better? It should be collaborative, multi-disciplinary.” (Physician, Private hospital, In-depth interview)*

However, participants also recognized that commitment and governance are both vital to the development of a collaborative health system. Furthermore, strong leadership and decision-making support were seen as necessary to prioritize the care of older adults and to develop policies and guidelines to partner across professionals and institutions.

*“There should always be coordination, interdisciplinary cooperation between doctors, non-doctors, ancillaries, and primary care, but it all boils down with the government. To maximize the limited budget, we can focus together on the preventive aspect (i.e., improvement of nutrition interventions at the community level) to get at least 30% improvement of wellness among older persons. We already have a national law (Republic Act No. 11223: Universal Health Care Act of 2019) and we need to beef it up by involving everyone including community health workers. The only thing that can further improve the health and welfare of older adults are not the private enterprise, not our society, but the government.” (Physician, Private hospital, In-depth interview)*

Most of the participants, specifically those in key roles in care facilities, desired an integrated health and social care system, which they believed would positively impact both service providers and recipients, but would require strong leadership and governance for its development.

## Discussion

**Fig. 1:**
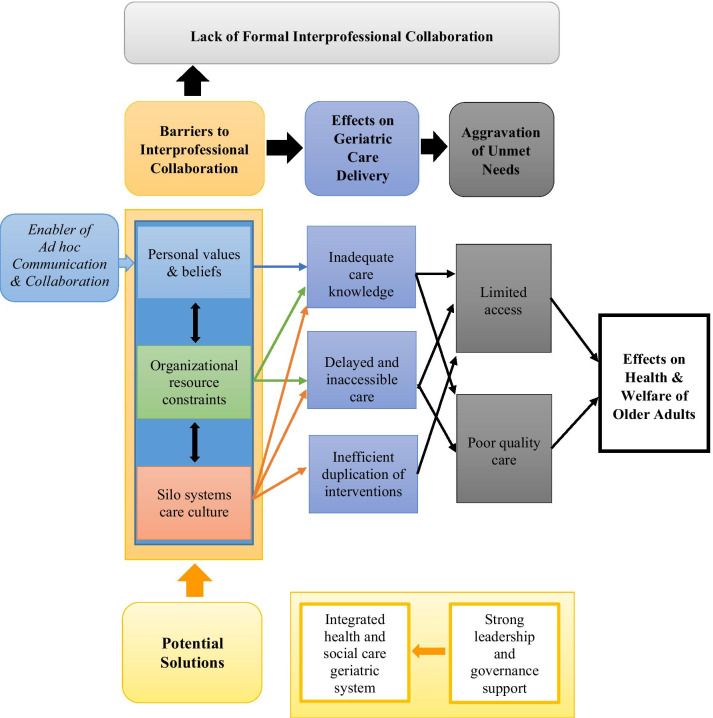
The status of IPC in the Philippines, barriers of IPC and impact on service provision and the unmet needs of older adults, and potential solutions

This study highlighted practitioners’ evaluation of the current state of teamwork and collaboration existing across various personnel, organizations, and healthcare levels in selected areas in the Philippines and the current barriers to IPC implementation in these settings. Formal IPC was believed to be primarily limited by systems issues, particularly a divided health and social care structure. System silo also impeded the provision of health and social welfare services for older adults exacerbating their unmet needs, as identified by participants. Several participants suggested, however, that these limitations could be overcome by strong centralized governance and key leaders committed to collaboration (Fig. 1).

Despite the need for integrated communication across sectors, ad hoc collaboration happened intermittently within individual institutions. Collaboration occurred only when individual staff members felt positive about collaborating but tended to focus primarily on general administrative concerns. This result is also similar among health professionals in Indonesian health centers identified to have limited interprofessional interactions [38]. This highlighted that different practitioners and institutions function disparately rather than collectively. This suggests that IPC is a human resource innovation not yet commonly practiced in selected areas in the Philippines even a national policy adopting a comprehensive geriatric service delivery has recently been enacted [5].

Although identified as an enabler of ad hoc communication practices, some personal values and beliefs such as deference for professional hierarchies, lack of mutual trust and respect, and willingness to collaborate were recognized as barriers to IPC, buttressing previous research results [9, 17, 39]. However, study participants frequently cited organizational barriers, such as resource constraints, posed greater barrier compared to interpersonal characteristics. This finding may indicate that IPC is not yet viewed as an approach to practice to increase the effectiveness of health care services provided [39], but rather an added responsibility requiring financial incentives, time, or other perceived needed arrangements. These were commonly observed in hospital interviews and similar from an Asian hospital study that identified cooperative relationship among health professionals was rated as a less frequent behavior commonly due to time-related issues [40]. In contrast, primary care workers were particularly interested in enhancing their professional roles and comfortable in doing collaborative practice [41].

The presence of organizational resource constraints affecting IPC practice could also be attributed to health system fragmentation in the Philippines wherein siloed health and social welfare organizations compete for limited resources. These generally siloed systems may push individuals and organizations to emphasize local and immediate concerns over coordinating, collaborating, and sharing resources with others to provide comprehensive elderly care [42]. This is contrary to the practice in other countries, such as Sweden and Japan where health systems are more integrated and operate with ample resources in an effort to provide high quality of care for older adults [43, 44]. The focal challenges in such settings foster trust, confidence, and respect among peers [45, 46]. Both individual and organizational siloes affecting IPC practice also exists due to disparate parameters set by professionals existing scope of practice and guidelines overseeing each organizations. These boundaries must be considered and amended to employ IPC as a policy solution that can potentially address siloes towards geriatric care improvement. Further, even as systemic siloes were perceived as the greatest challenge of collaborative practice, the three identified group of barriers in this study did not exclusively affect IPC implementation. They are rather related and impacting one another illustrating the complexity of IPC approach. IPC is similarly applied within organization, between organizations, and between professions with mutual relationships, structure, authority, shared responsibility, accountability, resources, and rewards [16]. Therefore, the health and social care system must focus on developing shared goals, indicators, activities, and task sharing in order to function as a cohesive network to drive a sustainable collaborative geriatric care delivery.

This study distinctively accentuated the existing barriers of IPC implementation and how it affected the quality of health and social care service delivery for older adults. In the Philippines, a decentralized healthcare system designed to improve decision-making and accountability at the local level is currently characterized by a fragmented public and private care delivery systems which may contribute to decreased quality, quantity, and efficiency of essential services [47, 48]. In other similar resource-limited settings, system fragmentation also resulted in diminished capacity, including duplication of activities and administrative inefficiencies [49]. These resulting deficiencies along with insufficient care knowledge among staff, inadequate awareness of the varying roles of organization may lead to confusion and competition, and consequently, delay care accessibility. These were observed in this study to negatively influence the overall quality of service delivery and in turn, were perceived to exacerbate the unmet needs of older adults. Yet, there remains little empirical evidence demonstrating the effect of such systems barriers on collaboration [17], and even less attention to how these limitations may negatively impact health outcomes of older adults in LMICs. The study findings justify the need to realize and implement the features of IPC practice eventually forging it as a solution integral in enhancing the quality of care for older Filipinos, along with other vital health service delivery approaches. Inequitable care access particularly among poor older people due to economic, transport, social, and care environment challenges had worsen their physical conditions [50], while wealthier older adults were observed to have greater capacity to acquire care services despite the barriers identified in this study. Hence, to address social inequity in geriatric care, patient targeting must be conducted together embedding IPC within and across organizations to promote better care access and break the silo. Addressing the identified barriers of collaboration and executing a team-based care, has been particularly effective at promoting equitable ease of access, integration of services, and continuity of care in countries of similar context as the Philippines [51].

In several studies, the benefits of collaboration have been shown to yield improved health services and outcomes such as increased efficiency, responsiveness, innovation, and creativity on the part of health workers [16, 24, 52, 53]. Likewise, in this study, all participants recognized that the integration of health and social care delivery systems is necessary to overcome current challenges in IPC implementation. However, addressing the personal values and beliefs of health practitioners might be easier to accomplish compared to solving systems siloes, which is more difficult to address. Our results showed that health and social service staff believe integration and communication can be facilitated by improved governance of key leaders with a clear commitment to collaboration. This finding aligns with previous evidence from both low- and high-income countries which emphasized the importance of strengthening governance and accountability at the system level to implement integrated care for older people [54]. Other research demonstrates that barriers to IPC can be addressed by forging shared accountability, interdependence, and mutual understanding across individuals and organizations [55].

To develop and institutionalize an integrated collaboration among health and social care in the future, enactment of policies, guidelines, and processes for collaborative practice arising from strong leadership and governance is necessary. Although collaboration in health care teams can be built in a voluntary basis and an interpersonal process requiring willingness and skills to be successful [17], IPC is a practical arrangement and approach requiring supportive structural components and working environment. The individual, organizational, and systemic determinants are important for effective collaboration and should not be treated separately [17].

The lack of formal IPC practice identified in this study also underpin the views that IPE is indispensable to prepare a collaborative practice-ready workforce that will respond to local needs [14]. Therefore, the academic curricula of health and social worker students must be restructured to initiate learning about, from, and with each professions and cover IPE topics and modalities on collaborative practice. Further, development and implementation of standardized in-service IPE programs to emphasize and instill a culture of collaboration among health and social workers employed in various health care settings must be sought. Pre-service and in-service interprofessional learning must also include concepts and discussions on personal determinants of collaboration to encourage individuals to deepen its insight, appreciation, and achieve a shared goal with other professions particularly that this determinant was identified to facilitate informal communication in the study. Incentives to encourage regular performance of individual and organizational collaboration strategies must also be in place to facilitate and sustain collaboration within the system. Further, to implement IPC as an effective policy approach, aside from training and education, restructuring the working environment, securing shared accountability, and team-based evaluation on collaboration must be conducted. Accomplishing these steps will allow the health and social systems to begin to collaboratively address the complex care needs of older adults and also assist in the acceleration of UHC implementation in the Philippines.

The results of this study point to the need for future in-depth exploration of the experiences of older adults and their unmet needs as identified by patients, their families, and communities related to IPC. In addition, further research in rural or disadvantaged localities is needed to determine the diversity of such experiences. Lastly, crafting appropriate and meaningful metrics will assist in charting national trends related to the effect barriers to IPC implementation that may impact the wellbeing of older adults.

The results of this study must be considered in relation to its strengths and limitations. The primary strength of this study is its wide coverage of both health and social service facilities and provider types, ranging from physicians, nurses, and other health professionals but also including community health workers and community leaders resulting in a larger number of interviews conducted enriching the results of this study. The majority of previous research on IPC covered only limited professionals working within a specific health facility and conducted minimal interviews [9, 25, 45, 46]. In addition, during the analysis stage, feedback on results was sought from study participants and various professionals on the research team, increasing reliability. Limitations to this study include the narrow geographic coverage as only two cities located within the main island of Luzon were part of the research design to demonstrate emerging patterns and barriers of IPC across two cities in the Philippines. The results may not be the same for, or representative of, other localities especially rural and geographically isolated and disadvantaged areas (GIDAs), even within the Philippines which is characterized by a wide range of social diversity. In addition, qualitative interviews focused on IPC and care for older adults and the experiences and perceptions relayed by participants may have been different if other health programs or populations were considered. Moreover, primary care level facilities and staff outnumbered practitioners from hospitals and nursing homes which may have resulted in over representing responses from those participants.

## Conclusion

This study provides evidence for the need for interprofessional teamwork and collaboration among health and social care practitioners to facilitate high-quality care for older adults. Several barriers were identified. Foremost was care systems siloing between different personnel, organizations, and care settings restricting formal IPC implementation. This barrier, along with personal values and beliefs and organizational resource constraints, were perceived to adversely affect geriatric service delivery, worsening the quality of life and health outcomes of older adults. IPC was felt as an additional task requiring financial motivations to be performed and existing silo increases health inequity. Strong and committed governance support was viewed essential to forge an integrated collaborative structure that can effectively render an inclusive and holistic care approach. These findings also highlight the impact of limited IPC that could assist in contextualizing importance of collaborative practice as an effective method in geriatric care delivery, including its potential solutions towards successful implementation. The results of this study is an appeal to adopt interprofessional education and collaboration as an approach to practice and realize its potential benefits in resource-limited countries such as the Philippines. Further studies are needed to evaluate this human resource intervention moving forward.

## Supplementary Information

**Additional file 1.** Interview guide questions. Semi-structured topic guide questions used during focus group discussions and in-depth interviews among health and social care workers. 

## Data Availability

The transcripts of all interviews conducted will not be made publicly available in the interest of protecting participants’ confidentiality, elaborately explained and stipulated in the agreement section of the informed consent form. Some selected illustrative quotes have been published to support the analysis.

## Abbreviations

IPC: Interprofessional collaboration
HRH: Human resources for health
UHC: Universal health coverage
LMICs: Low- and middle-income countries
WHO: World Health Organization
IPE: Interprofessional education
FGD: Focus group discussion
GMA: Greater Manila Area
ADL: Activities of daily living
OSCA: Office of the Senior Citizen Affairs
GIDA: Geographically isolated disadvantaged area
