# Supplementary material for: Interprofessional collaboration and barriers among health and social workers caring for older adults: a Philippine case study
Source: Hum Resour Health. 2021 Apr 19;19:52. doi: 10.1186/s12960-021-00568-1 (PMC8056548; doi:10.1186/s12960-021-00568-1)
Supplement: Supplementary file 1 — Additional file 1. Interview guide questions. Semi-structured topic guide questions used during focus group discussions and in-depth interviews among health and social care workers. [file 12960_2021_568_MOESM1_ESM.docx]

**Additional File 1. Interview guide questions**

| **Introduction** | |
| --- | --- |
| 1. How long have you been working in this facility? 2. Please describe the nature of your work  - *What are your roles / responsibilities in your institution?* |  |
| **Needs of Older Adults** | |
| 1. In your health facility, what are the generally observed characteristics of older adults in terms of the following:  - *Demographic characteristics (i.e., age, gender, education, type of residence)* - *Socio-economic characteristics (i.e., income status, current work)* - Presence of *social support* *(i.e., marital status, presence of relatives/friends or alone)* - *Presence of diseases (i.e., non-communicable and communicable diseases and other co-morbidities)* - *Limitations in activities of daily living and physical activity* - *Manifestation of mental difficulty* |  |
| 1. What are the observed health / medical or social care needs of older adults seen in your facility? 2. What are their usual sources of payment *(i.e., out-of-pocket, PhilHealth, or private insurance)?*  - *Ability for payment (i.e., ability to pay for healthcare services)* |  |
| **Programs/Services for Older Adults** | |
| 1. Are there programs / services that you provide for older adults? If YES, what are these?  - *What are the goals and objectives of these programs / services?* - *Who are the target recipients of these programs / services?* - *How are these programs / services delivered to recipients?* |  |
| 1. Are the programs / services provided for FREE or do they have a cost? If there is a fee involved, how much?  - *Do you think that the fee being asked are affordable? Why or Why not?*  1. Are there any partner organizations / institutions / referral units in the implementation of your programs / services? If YES, who are they?  - *How do these partners coordinate with your facility?* - *What are the roles and responsibilities of these partners?* |  |
| **Awareness on Gaps between Needs of Older Adults and Services Provided** | |
| 1. What are your perceptions of the programs / services provided for older adults?  - *Do you think you were able to respond to their needs? Why or why not?* - *Do you think that these programs / services are relevant to the needs of the older adults? Why or Why not?* |  |
| 1. Should these programs / services currently implemented be continued and enhanced or terminated? Why or why not? |  |
| 1. What are the strengths of the programs / services that you are providing? 2. What are the perceived facilitating factors in their implementation? |  |
| 1. What are the weaknesses of the programs / services that you are providing? 2. What are the perceived barriers / hindering factors in their implementation? |  |
| **Working and Training Experience of Personnel in Addressing Needs of Older Adults** | |
| 1. Prior to working in this facility, did you have experience in working with older adults? If YES, Where? 2. What is the relevant training and qualifications that you have received for the purpose of addressing the needs of older adults?  - *Where did you receive this training?* - *Does your current organization/institution also provided or assist you to have such training to improve your capability?*  1. Do you think the training was able to equip you with the knowledge and skills needed to perform your job? 2. What are your perceptions of the training received? |  |
| **Training Needs of Health / Social Care Professionals** | |
| 1. What do you think is the specific training that you need in delivering care for older adults?  - *Where do you think should trainings in handling older adults be provided, before working or while already employed in the facility?*  1. What do you think are the specific competencies that you need in delivering care for older adults? 2. What do you think should be the desirable experiences and characteristics of health workers addressing needs of older adults at the following levels:  - *Primary care,* - *Hospital,* - *Nursing home?* |  |
| **Working with other Health / Social Care Professionals**  *Note: To determine how much experience and the extent of interaction / collaboration that participants have with the following care professionals: physicians, nurses, rehabilitation therapists, nursing assistants, social workers, nursing home caregivers, and (if interview is at the primary care level) community health workers.* | |
| 1. Did you experience working or communicating / interacting with other health / social care professionals in addressing the health needs of older adults? If YES, which health care professionals do you commonly interact with? When and how does it usually happen?  - *Are there any formal / regular meetings with multiple professionals? Who leads or facilitates the meetings?* - *In addressing the needs of older adults, do you think that regular interaction and collaboration with health/social care workers should be conducted? Why or Why Not?*  1. What are your perceptions of interaction / collaboration with other health / social care professionals? Is it necessary and are you comfortable with it? Why or why not? 2. Do you think communication / interaction with other health / social care professionals enhanced your skills/competencies in addressing needs of older adults? If YES, in what way? If NO, why not? 3. Do you think communication / interaction with other health / social care professionals improved the health status of older adults? If YES, in what way? If NO, why not? 4. Did you receive formal or informal training or lecture in terms of communication / collaboration with other health / social care workers? If YES, where did you receive it? Is it specific in addressing the needs of older adults or not?  - *Do you think such training / lectures assisted you towards provision of care and services for older adults?* - *Should health / social workers undergo training / lectures on how to interact and collaborate towards addressing the needs of the clientele? Why or why not?*  1. How do you think the interaction would increase your understanding of the roles and responsibilities of other disciplines? 2. What are the perceived strengths or facilitating factors of working with other health / social care professionals in addressing the needs of older adults? 3. What are the perceived weaknesses or challenges / hindering factors of working with other health / social care professionals in addressing the needs of older adults? |  |
| **Recommendations** | |
| 1. Do you have any program or policy recommendations to promote effective health and social welfare services and improvement of interaction / collaboration with other health care workers to address the needs of older adults? |  |
